# State Medical Association Matters

**Published:** 1904-12

**Authors:** 


### TEXAS MEDICAL JOURNAL.

ESTABLISHED JULY, 1885.

PUBLISHED MONTHLY.—SUBSCRIPTION $1.00 A YEAR.

VOL. XX. AUSTIN, DECEMBER, 1904. No. 6.

### STATE MEDICAL ASSOCIATION MATTERS.

### Legislative Conference at Austin November 14th and 15th.

The meeting of the Committee on Public Policy and Legislation of the State Medical Association of Texas convened in the rooms of the Austin Club at 11 o'clock a. m., November 14th, at Austin, Texas. A call had been issued for delegates from each county society, and for the Councilors to meet with the committee in an advisory capacity for the purpose of considering needed medical legislation.

The meeting was called to order by President Daniel. Among those present were: Drs. R. H. Harrison, Columbus; W. B. Russ, San Antonio; W. S. Carter, Galveston; T. E. Stone, Jasper; Holman Taylor, Marshall; F. A. Painter, Pilot Point; W. D. Finney, Wrightsboro; R. L. Combs, Cooper; J. F. Bunkley, Seymour; Jno. V. Spring, San Antonio; M. E. McClure, Alto; J. P. Barton, Mooreville; W. F. West, Waxahachie; I. C. Chase, Fort Worth; F. Paschal, San Antonio; F. E. Daniel, Austin; M. M. Smith, Austin; T. J. Bennett, Austin; G. B. Foscue, Waco; B. F. Calhoun, Beaumont, and G. R. Tabor by special invitation.

Dr. Daniel stated the object of the meeting to be, first, to consider an anatomical bill, the passage of which the Committee on Public Policy and Legislation had been requested to secure by the House of Delegates; second, amendments to the Practice Act recommended for passage by the Board of Medical Examiners, and likewise recommended for passage by the House of Delegates;

---

*Reported for TEXAS MEDICAL JOURNAL by the Secretary.

third, the bill for a State Board of Health. Concerning this bill, Dr. Daniel stated that the Committee on Public Policy and Legislation at its meeting, in Dallas recently, decided to postpone the presentation of any bill for a State Board of Health for two years, because the text of the bill had not been decided upon and approved by the House of Delegates. The Constitution of the State Medical Association directs that "the Committee on Public Policy and Legislation, under the direction of the House of Delegates, shall represent the Association in securing and enforcing legislation in the interest of public health and of scientific medicine." In view of the many disagreements in the profession regarding previous bills, it was decided that more explicit directions from the House of Delegates was necessary, as well as a more intelligent understanding of the bill on the part of the profession. The political outlook was such that it was deemed advisable to attempt to secure more friends in the Legislature at a time when the State treasury was less depleted for the passage of such a bill. Dr. Daniel said in regard to the need of a Board of Public Health that this need is imperative. That the public's health interests are paramount. That in Texas such vital interests are less guarded than any other. That our progress is slow in comparison with Japan. He quoted Dr. Seaman, Surgeon U. S. A., as authority that on account of efficient oversight among 1000 Japanese wounded at Tokyo in one hospital there had not been a single death. The Japanese have an elaborate sanitary system. Medical inspectors accompany all branches of the army and inspect all food and water, and arrange the location of every camp. They have no trouble with embalmed beef nor fermenting vegetables, nor bad water, and expect to put more men in the field by maintaining a greater efficiency. The results have been marvelous and in striking contrast to our own statistics in the Spanish war, where for every 100 dying of wounds 1400 died of disease. Care of the public health is a science, and it pays the country that regards it. At present the power of quarantine is vested in the Governor, a man unskilled in sanitary matters. Responsibility for advice and action is vested in the State Health Officer. The physical welfare of the millions in Texas devolves upon one man, a responsibility too great to be vested in one individual no matter how efficient. The present laws authorize quarantine and inspection intended to keep disease out of the State. There are but meager provisions for controlling infectious diseases within our borders. Under present laws State and county authorities are in conflict. The proposed board should have power to prepare and enforce a sanitary code for Texas, and prepare and enforce under penalty rules and regulations and ordinances of a general nature for the improvement of hygienic and sanitary conditions of the State. Such a code should provide for the land and maritime quarantine regulations, for the reporting and management of infectious and contagious diseases; should regulate the reporting and tabulating of vital and mortuary statistics, provide regulations and facilities for vaccination, regulate the carriage of dead bodies and the adulteration of food and the selling of patent and harmful medicines, govern sanitation and require inspection of public buildings and railway trains, and many other features. In fact, should cover every phase of sanitation.

Dr. R. H. Harrison, of Columbus, was called on and stated that he had introduced a bill for a State Board of Health as early as 1876, and that it came very near passing, but died on the day of adjournment. He thought a board of health was very necessary for the State. That it contemplated the wisest and most efficient demonstration of sanitary rules at the least public expense. He believed personal enforcement was always very expensive. As an example, the attempted exclusion of bubonic plague from Texas recently cost the State $40,000. The yellow fever epidemic of last year proved also very expensive. He believed a board of health was cheaper. There is much need for a regulation by the State of lesser matters that could not well be included in special legislation. He believed that the present use of drugs and intoxicants needed regulation, and were to blame for much of the insanity. He favored turning over the guarding of our borders to the United States government, limiting the powers and duties of the State Board of Health to internal requirements.

Dr. Frank Paschal, of San Antonio, discussing the subject, stated that he favored in place of a State Health Officer a State Board of Health to consist of three physicians, one lawyer and one engineer. The continuance of the present quarantine system had previously depended upon the argument of efficiency. It was claimed that our system had been more efficient than one under control of a State Board of Health. This argument had been exploded by the recent yellow fever epidemic. There is greater safety in numbers of councilors for quarantining all localities than under rules and regulations laid down by one man, which are never as wise as those controlled by a wise board.

Adjourned until 2 p. m.

### AFTERNOON SESSION.

The meeting was called at 2 p. m. Dr. W. S. Carter, of Galveston, presented an anatomical bill, the text of which is as follows:

### A BILL

TO BE ENTITLED

An Act for the promotion of medical science by the distribution and use of unclaimed human bodies for scientific purposes through a board created for that purpose, and to prevent unauthorized uses and traffic in human bodies, and to legalize dissections and experiments by authorized persons.

*Be it enacted by the Legislature of the State of Texas:*

SECTION 1. That the professor of anatomy and the professor of surgery of each of the medical schools or colleges now incorporated, and the several medical and dental schools and colleges which may hereafter be incorporated in this State, shall be and are hereby constituted a board, to be known as the Anatomic Board of the State of Texas, for the distribution and delivery of dead human bodies hereinafter described, to and among such institutions and persons as, under the provisions of this act, are entitled thereto. The professor of anatomy in the Medical Department of the University of Texas at Galveston shall call a meeting of said board, for organization, at a time and place to be fixed by him, within thirty days after the passage of this act. The said board shall have the power to establish rules and regulations for its government, and to appoint and remove proper officers, and shall keep full and complete minutes of its transactions, and records sufficient for identification shall also be kept under its directions of all bodies received and distributed by said board and of persons to whom the same may be distributed, which minutes and records shall be open at all times to the inspection of each member of said board and of any district attorney or county attorney of this State.

SEC. 2. All public officers, agents and servants, and all officers, agents and servants of any county, city, town, district or other municipality, and of any and every alms-house, prison, morgue, hospital or any other public institution having charge of or control of dead human bodies required to be buried at public expense, are hereby required, after notification in writing by said board of distribution, or its duly authorized officers, or persons designated by the authorities of said board, then and thereafter to announce to said board, its authorized officer or agent, whenever such body or bodies come into his or their possession, charge, or control, and shall without fee or reward greater than the value of such fee as may be paid in any county, city, town or municipality, at the time of the enactment of this law for the burial of pauper bodies, deliver such body or bodies, and permit and suffer the said board and its agents and the physicians and surgeons, from time to time designated by them, who may comply with the provisions of this act, to take and remove all such bodies to be used within this State for the advancement of medical science; but no such notice need be given, nor any such body be delivered, if any person claiming to be and satisfying the authorities in charge of said body that he or she is of kindred or is related by marriage to the deceased, shall claim the said body for burial; but it shall be surrendered for interment; nor shall the notice be given for the body to be delivered if the deceased died of contagious disease, save tuberculosis or syphilis; nor shall the notice be given or the body be delivered if such deceased person were a traveler who died suddenly, in which case the said body shall be buried. It is further required that due effort be made by those in charge of such alms-house, prison, morgue, hospital, or other public institution having charge or control of such dead human bodies, to find kindred or relation of the deceased and notify him or her of the death, and failure to claim such body by kindred or relation within twenty-four hours after receipt of such notification shall be recognized as bringing such body under the provisions of this act, and delivery shall be made as soon thereafter to said board, its officers or agents, as may be possible. In case a body is claimed by relatives within ten days after it has been delivered to an institution or person entitled to receive the same under the provision of this act, it shall be delivered to them for burial after such institution or individual has been reimbursed by the relatives for expenses actually incurred in the transportation and embalming of said body.

Sec. 3. The said board, or their duly authorized agents, may take and receive such bodies so delivered as aforesaid and shall, upon receiving them, distribute and deliver to and among the schools, colleges, physicians and surgeons aforesaid in the manner following: Those bodies needed for lecture and demonstration of the said incorporated schools and colleges shall first be supplied, the remaining bodies shall then be distributed proportionately and equitable, the number assigned to each to be based upon the number of students receiving instruction or demonstration in normal or morbid anatomy or operative surgery, which number shall be certified by the dean of each school or college to the board at such times as it may direct. Instead of receiving and delivering said bodies themselves through their agent or servant, the said board may from time to time, either directly or by their designated officer or agent, authorize physicians and surgeons who shall receive them, and the number which each shall receive.

SEC. 4. The said board may employ public carriers for the conveyance of said bodies, which shall be well enclosed in suitable encasement and carefully deposited free from public observation. Said carrier shall obtain a designation receipt by name, or, if the person be unknown, by description of each body delivered by him, and shall deposit said designation with the duly authorized officer of said board or his duly authorized agent, receiving an acknowledgment thereof, which shall be transmitted to the sender and filed by him for reference.

SEC. 5. Any and all schools, colleges and persons who may be designated by said Anatomical Board of the State of Texas shall be, and are, by this act authorzed to dissect, operate upon, examine and experiment upon such bodies hereinafter described and distributed for the furtherance of medical science; and such dissections, operations, examinations and experiments shall not be considered as amenable under any already existing laws for the prevention or mutilation of dead human bodies. Such persons, schools or colleges shall keep a permanent record, sufficient for identification of each body received from such Anatomical Board or its agent, which record shall be subject to inspection by the board or its authorized officer or agent. The said Anatomical Board shall also have power to authorize incorporated schools or colleges and individual physicians and surgeons to experiment on the lower animals under bond hereinafter designated.

SEC. 6. No school, college, physician or surgeon shall be allowed or permitted to receive any such body or bodies until bond shall have been given to the State by such physician or surgeon, or by or in behalf of such school or college, to be approved by the clerk of the county court in and for the county in which such physician or surgeon may reside, or in which such school or college may be situated, and to be filed in the office of said clerk, which bond shall be in the penal sum of one thousand dollars, conditioned that all such bodies which the said physician or surgeon, or said college, shall receive thereafter shall be used and all such experiments on lower animals shall be conducted only for the promotion of medical science; and whosoever shall sell or buy such body or bodies, or in any way traffic in the same, or shall transmit or convey or cause or procure to be transmitted or conveyed said body or bodies to any place outside the State, shall be deemed guilty of misdemeanor, and shall on conviction be liable to a fine not exceeding two hundred dollars for each offense, or be imprisoned for a term not exceeding two years.

SEC. 7. Neither the State nor any county, nor municipality, nor any officer, agent or servant thereof shall be at any expense by reason of the delivery or distribution of any such body, but all expense thereof, and of said board of distribution, shall be paid by those receiving the bodies in such manner as may be specified by said Anatomic Board of the State of Texas, or otherwise agreed upon.

SEC. 8. That any person having duties imposed upon him by the provisions of this act who shall refuse, neglect or omit to perform said duties or any of them, as hereby required, shall, on conviction thereof, be liable to a fine of not less than one hundred dollars nor more than five hundred dollars for each offense.

SEC. 9. No compensation other than actual traveling expenses shall be received for their services in this capacity by members of this board.

SEC. 10. That all acts and parts of acts inconsistent with this act be and the same are hereby repealed.

SEC. 11 The fact that the incorporated medical colleges of this State are unable to secure subjects for anatomic dissection, and their efficiency thereby impaired, create an imperative public necessity and an emergency, and therefore the constitutional rule requiring bills to be read on three several days is hereby suspended, and this act take effect from and after its passage.

On motion by Dr. Holman Taylor, of Marshall, the bill was endorsed as read, and referred to the Committee on Public Policy and Legislation, and the support of every delegate pledged for its passage.

The following communication from Dr. J. T. Wilson, of Sherman, was read:

"SHERMAN, TEXAS, November 12, 1904.

*Dr. F. E. Daniel, Austin, Texas.*

DEAR DR. DANIEL: I am very sorry to inform you that it will be impracticable for me to leave here tomorrow to attend the meeting of the committee. I had hoped to do so until today, but find that I can not do so, much to my regret.

Owing to an unusual press of professional engagements since I saw you, and a very severe cold contracted from exposure, I have been unable to finish the paper suggested by Dr. Chase.

I sincerely hope you will have a pleasant and profitable meeting; that the situation will be gone over and discussed with a harmonious result, and all unite in a determination to push with judgment and discretion the medical legislation it is thought best to present. I hope the amendments to the medical law will be taken up by the council and the necessity of their enactment be urged upon them, that they may bring them before the county societies with the request that they insist upon their passage by their members of the Legislature.

I think that it will be well too to present the subject of the Anatomical Bill to the committee for consideration, if you will allow me to suggest it as being an important measure, and there should be such a law in the State. Without it our schools are handicapped in a measure, and it presents an obstacle to medical study that should not exist.

I beg you will call the attention of the conference to the Pure Food Bill that will come up again in Congress next winter, and that each take an interest in and be ready to work for it when the time comes.

I will repeat what I said at Dallas, that it will be an inopportune time to present a bill for a Board of Health to the coming Legislature in January, for the reasons that I stated then. I feel quite confident that it would be defeated, and it would injure the cause for the future. But let us all get together on a bill for 1906, prepare a bill that will be a great benefit to the State, that the entire profession can unite upon, begin the campaign for next year, and as the reorganization continues professional influence will increase, and by an early organized effort the pressure brought to bear will be so great that it will be sure to pass. The work should be done before the primaries, and committees should be appointed to inaugurate the campaign. The candidate for Governor should be forced to state his position before the primaries, as well as candidates for the Legislature.

Please give my love to my friends, and present my regrets to them for my absence.

Very sincerely yours,

J. T. Wilson."

Dr. C. E. Cantrell could not be present and sent the following paper, which was read by the Secretary:

"GREENVILLE, TEXAS, November 12, 1904.

By request, I present to the profession what seems to me the best means of securing the necessary legislation to best guard the health and protect both the people and the medical profession against imposters and medical pretenders.

First. I will say that it is necessary for the regular profession itself to become convinced that such legislation is necessary.

Second. We must convince the people so far as may be, and the legislators especially, of the justice of our cause. To this end each member of the profession must become a moulder of sentiment in the community where he lives and works.

Third. The profession must become thoroughly organized and of one mind regarding the legislation necessary. Petty differences must be laid aside, and we must present a solid front when we appear before the Legislature. This can only be done by the Councilors on their regular visits to the county societies presenting the plans of the State Committee to the County Legislative Committee and the county societies, for we will find use for each member in counties where the representative in the Legislature is against us on any proposed legislation.

Fourth. We must have a committee on legislation that knows no such word as fail. If at first we don't succeed, we must try again. When the Legislature meets this time, Dr. F. E. Daniel and Dr. T. J. Bennett, of Austin, will represent us. When they find a member of the Legislature against us, if the members of the county societies can be depended upon to write or telegraph asking them to go to the representative of the committee to get an explanation of the laws asked for, we will find it much easier to convince them.

Fifth. It is the duty of the members of the medical profession to take an interest in the election of the legislative and executive officers of our State, and this interest must be manifested before the primaries, as the primary election is really the one that decides who shall serve the people in Texas. We should obtain promises from the different candidates to support the measures we aim to put before the Legislature before the candidate is nominated for office. When pledges are secured the profession should stand together and elect him, unless equal promises are secured from all candidates for the office in question, in which case there can be no difference.

It is a false doctrine that physicians should take no part in politics. On the contrary, doctors, as a rule, are men of more than ordinary ability, and with ability comes responsibility. In a special sense we are responsible for the enactment of laws to protect the sick in this free government of ours, where the spirit of freedom goes too far in some directions; free to go and free to come; free to cheat and free to be cheated; free to dose and free to be dosed; and free not to be dosed at all, if common sense is put aside and fanatics hold sway over the innocent and ignorant on medical subjects. It is time, gentlemen, for us to put our hearts and consciences into this matter.

Hoping to be forgiven for my shortcomings in the past, I am ready to put more energy into these things, if I can only have the co-operation of the better element of the profession. The above is what seems to me to be the best way to get legislation, and is practically the one outlined to us by Dr. J. N. McCormick, Councilor for the American Medical Association, and I herewith make my politest acknowledgments for the same.

Respectfully submitted,

C. E. CANTRELL."

This paper was referred, on motion, to the Publishing Committee.

The following amendments to the Practice Act were proposed by the State Board of Medical Examiners, and endorsed by the House of Delegates:

"SEC. 4. Amend by adding in the last line, after the words 'surgery and midwifery,' the following: 'The said boards shall each procure a seal, which shall be the official seal of the boards.'"

"SEC. 5. Amend by striking out the words 'the seal of the State of Texas,' in the last line, and by adding instead 'the seal of the board granting such license.'"

"SEC. 6. Amend by adding in line 22, after the word 'character,' 'and that he or she is a graduate of a reputable medical school of recognized standing, requiring for graduation attendance upon not less than three regular sessions of not less than six months each, in three separate calendar years.'"

"SEC. 8. Amend by adding in line 25, after the words 'receive a license from the Board of Medical Examiners of Texas to practice in this State,' the following: 'Provided, that the medical laws and examining boards of such States grant equal rights and recognition to the licentiates of the boards herein created, and provided that the applicant has not previously been rejected by one of the boards of this State.' "

"SEC. 8. Amend by adding after the word 'midwifery,' at the end of the section, the following: 'Sixth. Provided, that all persons changing their residences to another county shall have their diplomas and licenses registered with the district clerk of said county, with certificate of date of issue and previous record.' "

"SEC. 13. Amend by adding in the fourth line, after the word 'imprisonment,' the following: 'In the county jail,' and by striking out in the seventh line in the same section (13), after the word 'surgeon and midwife,' the following sentence: 'Provided, that the provisions of this act do not apply to persons treating disease who do not prescribe or give drugs or medicine,' and add instead the following sentence: 'Provided, that those persons treating disease who do not prescribe or give drugs or medicine shall be examined in all the branches provided for in this act, except chemistry, materia medica and therapeutics.' "

These amendments were endorsed as well as the following to Section 13, which was proposed by Dr. Cantrell to correct a weakness in the bill, which did not contain the words "in the county jail," and which also was intended to throw the burden of proof upon the physicians charged with illegal practice. At present under the law it devolves upon the prosecution to prove that the offender was not practicing prior to 1885. This amendment has been passed upon by legal authority and drawn up by Senator-elect Looney, of Hunt county. It was referred on motion by Dr. Holman Taylor to the Committee on Public Policy and Legislation, with the request that it be included, if possible, with the other amendments to be laid before the Legislature.

"Amend Article 3788, Section 13, so as to read: 'Any person who shall practice medicine, surgery or midwifery in this State in violation of the provisions of this act shall, on conviction, be fined for each offense not less than fifty dollars nor more than five hundred dollars, or by imprisonment in the county jail not exceeding six months, or by both such fine and imprisonment; and it shall not be lawful for him or her to recover by action, suit, motion or warrant any compensation for services which may be claimed to have been rendered by him or her as such physician, surgeon or midwife; provided, that in any prosecution for the violation of the provisions of this act, when the accused justifies under either the first or second clause of Article 3788, it shall devolve upon said accused to show, as the case may be, that he or she was practicing medicine in Texas prior to January 1, 1885, and complied with the laws of this State regulating the practice of medicine in force prior to the 22d day of February, 1901 (being the date of the passage of the original act).' "

A similar suggestion from Dr. G. B. Foscue, of Waco, endorsed by Dr. Geo. H. Carter, of Marlin, was similarly referred.

Dr. W. S. Carter, of Galveston, moved that this conference recommend to the Committee on Public Policy and Legislation that the amendment proposed in Section 6 be changed so that all candidates for examination by the State Board of Medical Examiners shall be required to be graduates of reputable medical schools of recognized standing, requiring attendance upon not less than four regular sessions of seven months each in four separate calendar years, such requirements to apply only to those who graduate after the passage of this act.

On motion by Dr. W. F. West this amendment was adopted, and referred to the Committee on Public Policy and Legislation.

On motion by Dr. W. F. West, the high standards of the State Board of Medical Examiners was unqualifiedly recommended and endorsed by the conference.

Dr. T. J. Bennett, of Austin, presented the following paper, which was received and referred to the Publishing Committee:

"November 14, 1904.

GENTLEMEN: Having been named to speak on the subject of the 'Duties of County Committees on Public Health and Legislation,' I may state first that the duty of such committees, as defined by the by-laws of the county societies, are 'to enforce and support the sanitary and medical laws of the State in this county; to co-operate with the Committee on Public Policy and Legislation of the State Association in all matters pertaining to legislation, and to prosecute quacks and medical pretenders in the county.'

This is a very important committee, and no member of a competent society should be appointed on it who is not willing to work. Through the work of this committee the profession at large expects to reap a material advantage not only from an improvement of the sanitary and public health laws of the county, but by removing those who are not qualified to practice medicine and establishing the knowledge among the people that there is a difference between an educated and licensed physician and a medical quack or pretender.

After receiving appointment the committee should have a conference and set about at once to get in hand the State laws affecting medical matters in the county, the special county laws, and if there are incorporated towns or cities in the counties to collect all ordinances that bear upon the public health. Then after thorough study and understanding of these laws and ordinances the committee should prosecute an itemized investigation to determine whether they are being enforced.

1. The law regulating the practice of medicine, a copy of which may be obtained from the Secretary of the State Board of Medical Examiners at Austin, should be studied and intelligent steps taken to enforce it in the county.

2. The law referring to contagious and communicable diseases, passed by the Twenty-seventh Legislature, should next have attention. The chief requirement of this law is as follows: Any physician who shall knowingly conceal any case of contagious disease, or who shall fail to report to the county or city health officer any case of contagious disease, of which he may have knowledge, shall, upon conviction, be fined in any sum not less than $25 nor more than $100. The diseases referred to in this law are smallpox, varoloid, varicella, typhus fever, yellow fever, Asiatic cholera, typhoid fever, scarlet fever, diphtheria, measles, whooping cough. The report is to be made at once to the county health officer, or, in case the disease occurs in a city of 10,000 population or more, the report may be sent to the city health officer.

3. The next law requiring attention is that referring to vital statistics. An examination of the records in the county clerk's office will show the births and deaths reported by the physicians and midwives of the county.

The special county laws and city ordinances can not be named in this connection.

It will be found on investigation that much of the enforcement of the sanitary laws of the counties depends upon the physicians themselves. To illustrate, I will refer to the vital statistics law as carried out in my own county (Travis) for fifteen months since the law went into effect. The county clerk has recorded within this time 608 deaths and 747 births. There are fifty-eight physicians in the county and two midwives. The population of this county is estimated to be 35,000 and should show a birth rate of something like 40 to the 1000 population. The action of the vital statistics gives only 17. There being sixty physicians and midwives in the county, it shows only five-sixths of a child reported each and every month, and would indicate that the obstetric practice was falling off. A like failure to report deaths is just as apparent as the failure to report births. A magnificent showing which attests the superior skill and ability of our doctors! In fact, the records in the county clerk's office indicate that a number of our most prominent practitioners have virtually retired from obstetric practice, and strange to say that in the other line of practice, immense as it is sometimes reported to be, they never lose a case.

I would suggest that the Committee on Public Policy and Legislation make at least two reports a year, giving in detail the workings of the above laws in the county, and where a persistent neglect on the part of physicians to comply with the different laws as relate to the communicable diseases and births and deaths than an example be made of some good fellow who won't get mad and who is able to pay the fine.

The committee on the subject of prosecuting quacks and medical pretenders should proceed cautiously but firmly. Every medicay practitioner in the county should be known, and when a new man comes in the committee should know his record as early as possible. The splendid index system as proposed by the State Association to be kept by each county secretary will show a record of the new man, and, if his ways are devious and hard to find out, a copy of the Practice Act should be sent to him. If he does not comply with the law, then have him indicted, and stand together.

The Committee on Public Policy and Legislation has an envious opportunity to sow good seed and reap abundant rewards of merited praise from their fellow practitioners. It is true the public sometimes may not understand the motives of the committee in prosecuting their efforts to better the public health and protect the people from medical imposters, but a few years of such missionary work will bring about a better relationship on these subjects.

The committee will receive definite instructions from time to time from the State Committee on Public Policy and Legislation as to proposed laws and amendments, and the plans by which effective work in the field of politics can be carried out, and these suggestions and instructions should be promptly reported to the next meeting of the society. The committee will meet with many rebuffs from politicians and will be the recipients of much advice from this source. When the earnest worker is getting behind the candidate before the primaries, the candidate is liable to suggest that he would rather see his preacher dragged into the slime and filth of politics than his doctor, but the doctor will know how to answer him and get behind him just the same. The time has passed when medical men can be poo-pooed out of their rights by any such sophistry. It is clearly the duty of this committee, backed up by the society, to take an active interest in all political matters pertaining to the public health. But I must stop here. The discussion of the plan of campaign has been given to another, the State Committee."

The following bill for the creation of a State Board of Health was then read by Dr. F. E. Daniel, being a revision of the original bill presented before the Legislature two years ago, intended to serve as a foundation for a bill to be adopted by the conference.

---

### TUESDAY—MORNING SESSION.

On motion by Dr. Calhoun, the consideration of the bill providing for a Board of Health was undertaken. The bill was read and discussed by sections. The text of the bill, as amended and endorsed and referred to the committee will be submitted to the Attorney General for an opinion as to constitutionality, and will then be published in the January number of the *Texas Medical Journal.* Reprints will be struck off and submitted to every county medical society in the State. At the April meeting of the Association at Houston the House of Delegates will pass upon it and instruct the committee.

A vote of thanks was extended Dr. Worsham for an invitation to visit the State Pasteur Institute, now in operation at the Austin Insane Asylum.

The Hepburn Pure Food Bill was endorsed by the conference representing the State and county societies, and the Secretary requested to correspond with Texas representatives and Senators, urging their support of this measure in the coming session of Congress.

Adjourned *sine die.*

